# Dopaminergic Dysregulation in Syndromic Autism Spectrum Disorders: Insights From Genetic Mouse Models

**DOI:** 10.3389/fncir.2021.700968

**Published:** 2021-07-23

**Authors:** Polina Kosillo, Helen S. Bateup

**Affiliations:** ^1^Department of Molecular and Cell Biology, University of California, Berkeley, Berkeley, CA, United States; ^2^Helen Wills Neuroscience Institute, University of California, Berkeley, Berkeley, CA, United States; ^3^Chan Zuckerberg Biohub, San Francisco, CA, United States

**Keywords:** dopamine, autism spectrum disorder, Angelman syndrome, Fragile X syndrome, Rett syndrome, PTEN hamartoma tumor syndrome, Tuberous Sclerosis Complex, genetic mouse models

## Abstract

Autism spectrum disorder (ASD) is a neurodevelopmental disorder defined by altered social interaction and communication, and repetitive, restricted, inflexible behaviors. Approximately 1.5-2% of the general population meet the diagnostic criteria for ASD and several brain regions including the cortex, amygdala, cerebellum and basal ganglia have been implicated in ASD pathophysiology. The midbrain dopamine system is an important modulator of cellular and synaptic function in multiple ASD-implicated brain regions via anatomically and functionally distinct dopaminergic projections. The dopamine hypothesis of ASD postulates that dysregulation of dopaminergic projection pathways could contribute to the behavioral manifestations of ASD, including altered reward value of social stimuli, changes in sensorimotor processing, and motor stereotypies. In this review, we examine the support for the idea that cell-autonomous changes in dopaminergic function are a core component of ASD pathophysiology. We discuss the human literature supporting the involvement of altered dopamine signaling in ASD including genetic, brain imaging and pharmacologic studies. We then focus on genetic mouse models of syndromic neurodevelopmental disorders in which single gene mutations lead to increased risk for ASD. We highlight studies that have directly examined dopamine neuron number, morphology, physiology, or output in these models. Overall, we find considerable support for the idea that the dopamine system may be dysregulated in syndromic ASDs; however, there does not appear to be a consistent signature and some models show increased dopaminergic function, while others have deficient dopamine signaling. We conclude that dopamine dysregulation is common in syndromic forms of ASD but that the specific changes may be unique to each genetic disorder and may not account for the full spectrum of ASD-related manifestations.

## Introduction

Autism spectrum disorder (ASD) is a neurodevelopmental disorder primarily characterized by deficits in social interactions, as well as repetitive, restricted, and inflexible behaviors (American Psychiatric Association, 2013). ASD is typically diagnosed early in life, although the clinical presentation and symptom severity can vary widely between individuals and across the lifespan. The latest CDC estimates suggest that 1 in 54 children are diagnosed with ASD by the age of 8, with ASD diagnosis being over four times more prevalent in boys than girls (Maenner et al., 2020).

The exact neurobiological basis of ASD remains unknown. This is in part due to a heterogenous behavioral presentation accompanied by widely divergent genetic, transcriptomic and epigenomic signatures found in individuals with ASD (De La Torre-Ubieta et al., 2016), although convergent molecular subtypes are being identified (Ramaswami et al., 2020). Studies in animal models and human subjects suggest that different brain regions and circuits contribute to distinct aspects of ASD symptomatology. Several brain regions implicated in ASD include the cortex (Donovan and Basson, 2017; Varghese et al., 2017), amygdala (Zalla and Sperduti, 2013; Donovan and Basson, 2017), cerebellum (Fatemi et al., 2012; Hampson and Blatt, 2015), and basal ganglia (Fuccillo, 2016; Subramanian et al., 2017). In particular, alterations in basal ganglia circuits may be a key driver of the restricted, repetitive behaviors in ASD (Fuccillo, 2016). Consistent with this, human neuroimaging studies have identified structural changes in the basal ganglia that correlate with repetitive behaviors (Asano et al., 2001). Increased striatal volume, the caudate nucleus in particular, has been identified in several studies (Hollander et al., 2005; Langen et al., 2009; Langen et al., 2014), while for other basal ganglia structures both volumetric decreases and increases have been reported (Estes et al., 2011; Schuetze et al., 2016). One study reported a decrease in ventral striatal volume in ASD subjects correlating with the severity of social deficits (Baribeau et al., 2019).

Dopaminergic projections originating from the midbrain exert wide-spread influence over multiple brain regions implicated in ASD pathophysiology including the basal ganglia, cortex and amygdala. Within these regions, dopamine (DA) serves as an important neuromodulator that fine-tunes cellular and synaptic function. Importantly, DA neurons and their projections are anatomically and functionally distinct. Dopaminergic input to the dorsal striatum originates primarily from the substantia nigra pars compacta (SNc; nigrostriatal pathway) and is fundamental to the context-appropriate, flexible selection of actions (Howard et al., 2017). In turn, ventral tegmental area (VTA) dopaminergic projections to the ventral striatum including the nucleus accumbens (mesolimbic pathway) are involved in reward processing (Watabe-Uchida et al., 2017), salience (De Jong et al., 2019), and motivation (Hamid et al., 2016; Mohebi et al., 2019). Cortical dopaminergic projections (mesocortical pathway) are sparser but provide important modulation of cognitive processes, including sensory gating and working memory (Ott and Nieder, 2019). Precisely controlled and appropriately timed release and clearance of DA at its targets is critical for adaptive behavior and learning. As discussed in other articles in this special issue, the dopaminergic system is highly heterogeneous. Recent gene profiling studies have identified several distinct sub-populations of DA neurons defined by their unique transcriptional signature (Poulin et al., 2014; La Manno et al., 2016; Hook et al., 2018; Kramer et al., 2018; Saunders et al., 2018; Tiklová et al., 2019; Poulin et al., 2020). Mouse models are being developed to allow intersectional genetic targeting of these newly defined dopaminergic populations to better understand their anatomical connectivity, functional properties and behavioral roles (Poulin et al., 2018; Kramer et al., 2021). Such approaches will enable a more fine-grained understanding of dopaminergic functions in both health and disease.

Given that DA is a key modulator of neuronal activity in several brain regions associated with ASD, there is compelling reason to ask whether dysfunctional DA signaling could impact brain activity across a range of ASD-implicated brain structures. The formally articulated dopamine hypothesis of ASD (Paval, 2017) postulates that functional dysregulation of DA projection pathways could contribute to the behavioral alterations that lead to ASD symptomology. For example, aberrant mesolimbic DA signaling could diminish the reward value assigned to social stimuli, eventually resulting in failure to acquire socioemotional reciprocity and/or communication skills. Alterations in mesocortical DA neurotransmission could translate to abnormal sensory processing and/or cognitive rigidity. Aberrant nigrostriatal DA signaling could promote stereotyped or repetitive motor movements and/or hyper-reliance on habitual behavioral control. Thus, the dopamine hypothesis of ASD as currently stated, predicts that either hyper- or hypo-dopaminergic signaling within target regions could lead to or exacerbate ASD-related behavioral changes (Paval, 2017). This is consistent with the idea of an “inverted U” shape to describe the optimal level of DA receptor signaling, wherein too much or too little DA are detrimental to cognitive functions (Arnsten, 1997; Cools and D’esposito, 2011). The DA hypothesis of ASD further predicts that aberrations at distinct projection targets could control discrete aspects of ASD symptomatology. Importantly, any changes in dopaminergic neurotransmission would likely lead to complex adaptations in downstream circuitry, involving pre- and post-synaptic alterations and plasticity.

In this review we will first summarize the evidence for dopaminergic involvement in ASD gleaned from human studies and then examine whether cell-autonomous changes in the DA system are commonly observed in animal models of ASD, with a focus on genetic mouse models. A particular question we aim to address is whether the wide diversity of genetic mouse models of ASD, with both constitutive (in which the mutation is present in all cells throughout development) and conditional mutations (in which the gene is altered in DA neurons only), show concordant changes in the function of midbrain DA neurons that may be causal for ASD-related behavioral changes. In other words, is there a consistent dopaminergic signature observable across different genetic causes of ASD?

For the purposes of this review, we will focus on the cell autonomous properties of DA neurons. Several important parameters determine the functionality of midbrain DA neurons at the level of the cell bodies, or somas, and axon terminals, typically examined within the striatum, which has the densest dopaminergic innervation. First, the number of DA neurons determines whether ASD-linked-mutations may cause DA neuron degeneration, or, in contrast, enhance the generation or survival of dopaminergic cells. Second, morphological properties such as cell size and dendritic architecture are examined to study structural aberrations that may impact the intrinsic excitability or connectivity of DA neurons. Third, changes in the firing properties of DA neurons can be assessed using electrophysiology or functional imaging techniques. Fourth, the levels of tyrosine hydroxylase (TH), the rate limiting enzyme in DA synthesis, determine overall DA production capacity and can be examined both in cell bodies and axon terminals by western blot or immunohistochemistry. DA production and storage determine the total tissue DA content in somas and axonal target regions, which is typically examined by high-performance liquid chromatography (HPLC). Finally, functional measures of basal (spontaneous) or evoked DA release by fast-scan cyclic voltammetry (sub-second timescale) or microdialysis (temporal resolution of minutes) are used to examine DA release and re-uptake in the midbrain somatodendritic region or axon terminal target regions.

### Evidence for Dopaminergic Involvement in ASD

#### Brain Imaging and Pharmacological Interventions in Individuals With ASD

Neuroimaging studies in individuals with ASD support the potential involvement of altered DA signaling, especially in striatum and prefrontal cortex, in the behavioral manifestations of ASD. For example, PET imaging of fluorodopa accumulation revealed reduced presynaptic DA levels in the prefrontal cortex of children with ASD (Ernst et al., 1997). In turn, PET imaging of radioligand binding to the dopamine active transporter (DAT) showed increased binding in the orbitofrontal cortex of high-functioning adults with ASD (Nakamura et al., 2010); however, changes in dopaminergic function at rest have not been detected universally (Makkonen et al., 2008). During task performance, a reduction in phasic striatal DA events evoked by social stimuli in children and adolescents with ASD have been reported in several studies (Scott-Van Zeeland et al., 2010; Zürcher et al., 2020). Together, these data support the involvement of functional changes in dopaminergic signaling in ASD. Yet, differences in experimental methodology and the inherent heterogeneity among individuals with ASD, including differences between age groups, also lead to conflicting results across studies and warrant further investigation.

Pharmacological manipulations of DA neurotransmission are currently used to manage some of the symptoms of ASD. Roughly 80% of children with ASD suffer from irritability (Mayes et al., 2011). Several dopamine receptor D2 subtype antagonists, such as risperidone and aripiprazole, have been successfully used to improve irritability, aggression and tantrum behaviors in individuals with ASD (Mccracken et al., 2002; Owen et al., 2009; Aman et al., 2010). However, current guidelines recommend against long-term and wide-spread use of these medications due to significant side effects and modest evidence for clinical efficacy (Leclerc and Easley, 2015; Howes et al., 2018). Nonetheless, meta-analyses of placebo-controlled randomized clinical trials do find significant, albeit modest in size, improvement in irritability and aggression scores in children and adolescents with ASD following short-term (<6 months) risperidone or aripiprazole treatment (Sharma and Shaw, 2012; Hirsch and Pringsheim, 2016). Secondary outcome measures and *post hoc* analyses further show a significant decrease in stereotypy scores with aripiprazole in children with ASD (Aman et al., 2010; Marcus et al., 2011).

Together, functional neuroimaging and pharmacological interventions in human subjects support dopaminergic involvement in ASD. However, evidence from human studies for a role of DA in core autism symptoms of reduced sociability and restricted, repetitive behaviors is correlational. In contrast, rodent models in which manipulations of DA signaling are possible conclusively demonstrate causal DA involvement in sociability, stereotypies, and other ASD-relevant manifestations (Presti et al., 2003; Gunaydin et al., 2014; Lee et al., 2018).

#### Genetic Evidence for Dopaminergic Involvement in ASD

ASD is a complex polygenic disorder, with SFARI gene currently listing 1003 genes implicated in autism, with 418 genes classified as high confidence or strong candidate ASD risk genes (^1^ database accessed 04/22/2021, version 2020 Q4). Strong candidate genes fall into two main categories based on the current SFARI classification. Those assigned ‘score 1’ are high confidence candidates and can be found on the SPARK gene list or on the list of genes reported by Satterstrom et al. (2020). These genes generally have at least three *de novo* likely gene-disrupting mutations reported in the literature. Genes assigned ‘score 2’ are strong candidates with two likely gene-disrupting mutations reported, implicated by GWAS replicated across different cohorts and supported by functional evidence. Both of these categories are therefore strongly supported by currently available published literature to play a causal role in ASD. However, the large majority of ASD-associated genes fall into the ‘score 3’ category based on a single reported mutation and either unreplicated association studies or rare inherited mutations. These ‘score’ metrics allow for consistent evaluation of the strength of evidence across different genes and have been widely adopted by researchers.

Polymorphisms and mutations in genes encoding proteins that directly control DA neurotransmission have been implicated in ASD (Nguyen et al., 2014). These include the genes encoding the dopamine active transporter (“DAT,” *SLC6A3*) (Hamilton et al., 2013; Bowton et al., 2014; Campbell et al., 2019; Dicarlo et al., 2019), plasma membrane monoamine transporter (*SLC29A4*) (Adamsen et al., 2014), several dopamine receptors (*DRD1*, *DRD2, DRD3, DRD4*) (Hettinger et al., 2008; De Krom et al., 2009; Reiersen and Todorov, 2011; Staal et al., 2011, 2018; Hettinger et al., 2012), and genes encoding proteins important for dopamine synthesis (*DDC*) (Toma et al., 2012) and catabolism (*MAO*, *COMT*) (Cohen et al., 2003; Yoo et al., 2009, 2013; Cohen et al., 2011; Verma et al., 2014; Wassink et al., 2014). Only *SLC6A3* is currently classified as a strong candidate ‘score 2’ gene, while others (*SLC29A4, DRD1, DRD2 DRD3, DDC, MAO*) fall into the ‘score 3’ category where causal involvement in ASD is supported only by suggestive evidence. Taken together, there is evidence that mutations in genes encoding key dopaminergic proteins may contribute to ASD risk, however, none of these currently rise to the top of the list of high confidence ASD-related genes.

Notably, there is overlap in the dopaminergic genes identified as altered in individuals with ASD with those found in individuals diagnosed with attention-deficit hyperactivity disorder, bipolar disorder, schizophrenia or obsessive-compulsive disorder (Khanzada et al., 2017). This suggests that while these disorders have distinct presentations, they may share some overlapping etiology, which could commonly involve aberrant DA signaling within basal ganglia and cortical circuits.

#### Dopaminergic Perturbations Can Cause ASD-Related Phenotypes in Mice

Several *SLC6A3* mutations identified in individuals with ASD have been modeled in mice. In a mouse model with a rare *Slc6a3* coding variant Val559, implicated in ASD in two unrelated male probands (Bowton et al., 2014), researchers observed increased basal striatal DA levels with *in vivo* microdialysis and a reduction in vertical motor behaviors such as rearing (Mergy et al., 2014). In a different model, with a threonine-to-methionine substitution at the 356 site in DAT (T356M) (Hamilton et al., 2013), researchers found reduced DA clearance rate concomitant with increased striatal DA metabolism and reduced DA synthesis (Dicarlo et al., 2019). Behaviorally, T356M mice showed hyperlocomotion, increased performance in the rotarod test and increased vertical rearing, resembling motor stereotypies. The mice also showed no preference for interacting with a novel mouse over a novel object, indicating reduced social approach behavior (Dicarlo et al., 2019). This study, in particular, demonstrates both construct and face validity of the *Slc6a3* T356M mouse for studying ASD-relevant changes in dopaminergic neurotransmission *in vivo*.

In general, pathogenic *SLC6A3* mutations, including those implicated in human ASD, are likely to increase the availability of extracellular DA due to reduced DAT function. This can happen either via impaired DA uptake and/or anomalous efflux leading to augmented DA signaling. In turn, increased DA signaling, either by reducing DAT expression in SNc neurons with siRNA or optogenetic stimulation of the nigrostriatal pathway, is sufficient to cause repetitive behaviors and sociability deficits in wild-type mice (Lee et al., 2018). Further, intra-striatal infusion of the D1 receptor antagonist SCH 23390 reduces naturally occurring motor stereotypies in a deer mouse model of ASD in the absence of general motor suppression (Presti et al., 2003). By contrast, in the ventral striatum, optogenetic suppression of the VTA-to-Nucleus Accumbens (NAc) (mesolimbic) pathway reduces sociability in mice, while activation increases social interactions via NAc D1 receptors (Gunaydin et al., 2014). Together, these studies suggest possible sub-region differences in DA dynamics contributing to distinct aspects of ASD-associated behaviors: hyperdopaminergia in the dorsal striatum could cause persistent and spontaneous stereotypies, while hypodopaminergia in the nucleus accumbens could lead to reduced sociability.

There are several unique features of the DA system that could support such distinct region-specific functional outcomes manifesting in ASD symptomatology. First, as a neuromodulator responsible for fine-tuning circuit function, either too much or too little DA signaling can cause aberrant information processing and behavior. The inverted U relationship between DA signaling and functional outcomes supports this interpretation (Cools and D’esposito, 2011). Second, the inherent heterogeneity of dopaminergic cell types based on projection targets, action potential properties and other parameters (Lammel et al., 2008), can support ‘independent’ functional outcomes within distinct brain regions through a combination of distinct cell intrinsic characteristics and local modulatory mechanisms (Sulzer et al., 2016). Third, the loop architecture of basal ganglia circuits could theoretically lend itself to either selective reinforcement or suppression of one dopaminergic domain by another (e.g., modulation of motor output by limbic domains) (Aoki et al., 2019; Lee et al., 2020). Finally, it should also be noted that compromised uptake or anomalous efflux of DA can augment dopaminergic catabolism thereby reducing DA recycling and increasing the burden of *de novo* DA synthesis, which over time could lead to reduced DA levels (Lee et al., 2018; Dicarlo et al., 2019). This, in turn, would compromise dopaminergic neurotransmission due to lack of neurotransmitter availability. Thus, an initial hyperdopaminergic state may over time evolve into deficient DA signaling. Consequently, it is possible that aberrations in DA neurotransmission in ASD are dynamic and evolve over time. In this respect, longitudinal studies conducted in humans with ASD and in mouse models would be helpful to our understanding of DA involvement in ASD pathophysiology across the lifespan.

### Genetic Mouse Models of ASD

Without explicit biomarkers available for ASD and diagnostic criteria based on complex behaviors, ASD-related phenotypes in mouse models can only be approximated. Typically, studies assess ultrasonic vocalizations, social preference, social recognition and social interaction as a proxy for communication and sociability deficits, and motor learning and motor stereotypies as a proxy for repetitive, restrictive behaviors (Kazdoba et al., 2015). Tasks assessing cognitive performance, cognitive flexibility, sensory perception and learning have also been utilized. Yet, with these measurements of behavioral outcomes it is difficult to distinguish between mouse models of ASD and those modeling other neuropsychiatric conditions, which frequently present with overlapping behavioral phenotypes. Thus, while functional studies indeed provide evidence for ASD-related mutations in ‘dopaminergic’ genes causing changes in DA metabolism, release, re-uptake, or pre/post-synaptic signaling, the specificity of the reported behavioral phenotypes to ASD could be questioned. In other words, observing changes in the DA system with mutations that directly affect DA neurotransmission is expected, but do other genetic causes of ASD also alter DA signaling?

To address whether there is a ‘dopamine signature’ in ASD, it can be useful to examine mouse models with mutations in high confidence ASD-risk genes. Several of these genes are associated with syndromic neurodevelopmental disorders, which also frequently present with epilepsy, intellectual disability and other medical and behavioral conditions. Angelman syndrome, Fragile X syndrome, Rett syndrome, PTEN hamartoma tumor syndrome, and Tuberous Sclerosis Complex are some examples of syndromic neurodevelopmental disorders associated with high rates of ASD. Genetic mouse models of these neurodevelopmental disorders are well-established and show some ASD-relevant behavioral phenotypes. These can include reduced social interaction, impaired cognitive performance, and perseverative or repetitive behaviors. Here we summarize studies that have directly examined dopaminergic function in these models. Table 1 provides a summary of the mouse models discussed and the main findings related to DA neuron structure and function.

**TABLE 1:** Summary of DA neuron phenotypes in mouse models of syndromic autism spectrum disorders.

Syndrome	Human gene	Mouse model	Gene manipulation	Type of mutation	Age at manipulation	Age at experiment	DA neuron number	DA neuron cell body morphology	DA neuron intrinsic excitability	DA neuron axonal morphology	DA release	TH expression	DA tissue content	DAT expression and function	References
Angelman	*UBE3A*	Ube3a m-/p+ and Ube3a flox/p+ x TH-Cre	deletion	constitutive and conditional	embryonic	P60-P90	–	–	Unchanged in VTA neurons	–	Unchanged evoked release in NAc (FCV, optogenetic stim)	–	–	–	Berrios et al., 2016
		Ube3a m-/p+	deletion	constitutive	embryonic	P210-240	Decreased SNc neuron number (IHC)	–	–	–	–	–	–	Increased striatal DAT expression (IHC)	Mulherkar and Jana, 2010
		Ube3a m-/p+	constitutive	deletion	embryonic	adult	–	–	–	–	–	–	–	Decreased DAT function (synaptosomal efflux)	Steinkellner et al., 2012
		Ube3a m-/p+	deletion	constitutive	embryonic	P90-120	No changes in SNc or VTA (IHC)	–	–	–	Decreased evoked release in dorsal striatum and increased in ventral striatum (*in vivo* FCV, MFB electrical stim)	No change in dorsal or ventral striatum (WB)	No change in dorsal or ventral striatum (HPLC)	–	Riday et al., 2012
		Ube3a m-/p+	constitutive	deletion	embryonic	P84-105	–	–	–	–	–	–	Increased in striatum and unchanged in midbrain (HPLC)	–	Farook et al., 2012
ASD	*15q11-q13*	7p duplication	duplication	constitutive	embryonic	P84-105	–	–	–	–	–	–	Increased in striatum and midbrain (HPLC)	–	Farook et al., 2012
Fragile X	*FMR1*	Fmr1 KO	deletion	constitutive	embryonic	adult	–	–	–	–	Increased evoked release in PFC and decreased in striatum (*in vivo* microdialysis, amphetamine stim)	–	–	–	Ventura et al., 2004
		deletion	Fmr1 KO	embryonic	constitutive	P28-42; P63-77	–	–	–	–	–	–	No change in young mice, increased striatal content in older mice (HPLC)	–	Sørensen et al., 2015
		Fmr1 KO	deletion	constitutive	embryonic	P28-31; P209-221	–	–	–	–	–	–	No change in striatum in young or old mice (HPLC)	–	Gruss and Braun, 2001, 2004
		Fmr1 KO	deletion	constitutive	embryonic	P70; P105-140	–	–	–	–	No change in evoked release in dorsal striatum in younger animals but decreased in older mice (FCV, electrical stim)	–	No change in striatum in young or old mice (HPLC)	No change in DAT function in young animals and reduced DAT function in older mice (FCV)	Fulks et al., 2010
		Fmr1 KO	deletion	constitutive	embryonic	P81-166	Decreased SNc and unchanged VTA neuron number (stereology)	–	–	–	–	No change in dorsal or ventral striatum (WB)	–	–	Fish et al., 2013
		Fmr1 KO	deletion	constitutive	embryonic	P56-112	–	–	–	Increased axonal complexity in striatum (IHC)	–	No change in midbrain (IHC), dorsal or ventral striatum (IHC, WB)	–	Decreased striatal DAT expression (WB)	Chao et al., 2020
Rett	*MECP2*	Mecp2-/y	deletion	constitutive	embryonic	P21; P56	–	–	–	–	–	–	No change in striatum or midbrain in juveniles or adults (HPLC)	–	Santos et al., 2010
		Mecp2-/y	constitutive	deletion	embryonic	P24; P35; P55	Decreased SNc cell number in adults (IHC)	Decreased SNc soma size in adults (IHC)	–	–	–	Decreased midbrain and striatal expression in adults (IHC)	No change in midbrain and decreased in striatum in adults (HPLC)	–	Panayotis et al., 2011a, b
		Mecp2-/y	deletion	constitutive	embryonic	adult	–	–	–	–	–	Decreased in midbrain (WB)	Decreased in striatum (HPLC)	–	Samaco et al., 2009
		Mecp2-/y	deletion	constitutive	embryonic	P28-35	–	–	–	–	–	–	Decreased in dorsal striatum, more in rostral than caudal striatum (HPLC)	–	Kao et al., 2013
		Mecp2fl/y x Dlx5/6-Cre	deletion	conditonal KO	embryonic	P28-35	–	–	–	–	–	No change in rostral striatum and midbrain, increased in caudal striatum (WB)	Decreased in rostral dorsal striatum, increased in caudal dorsal stratum, no change in middle striatum or midbrain (HPLC)	–	Su et al., 2015
		Mecp2-/y	deletion	constitutive	embryonic	P30-57; P101-105	–	–	Decreased membrane capacitance in SNc	–	–	–	–	–	Gantz et al., 2011
		Mecp2+/-	deletion	constitutive	embryonic	P16-30; P169-519	–	Decreased soma size and dendritic complexity in SNc (IHC)	Decreased membrane capacitance and increased resistance in SNc	–	Decreased evoked release in striatum of adult mice (FCV, electrical stim)	–	–	–	Gantz et al., 2011
PHTS	*PTEN*	Pten+/–	deletion	constitutive	embryonic	P56	–	–	–	–	–	Increased in striatum and PFC (IHC,WB)	–	–	He et al., 2015
		Ptenfl/fl x DAT-Cre	deletion	conditional KO	embryonic	adult	Increased SNc and VTA cell number (IHC)	Increased soma size in SNc (IHC)	–	Increased axon terminal size (IHC)	–	–	–	–	Inoue et al., 2013
		Ptenfl/fl x DAT-Cre	deletion	conditional KO	embryonic	P84-112	Increased SNc and VTA cell number (stereology)	Increased soma size in SNc and VTA and increased dendrites in SNc (IHC)	–	Increased axonal size in caudal striatum (IHC)	No changes in basal or evoked release in dorsal striatum (*in vivo* microdialysis)	Increased in midbrain and unchanged in striatum (IHC)	Increased in striatum and midbrain (HPLC)	No change in DA clearance rate (microdialysis)	Diaz-Ruiz et al., 2009
		Ptenfl/fl x DAT-Cre-ERT	deletion	conditional KO	8-10 weeks	P98-112	No changes in SNc (IHC)	Increased soma size in SNc and VTA (IHC)	–	Increased axon terminal density in striatum (IHC)	–	Increased in midbrain and striatum (WB)	Increased in striatum (HPLC)	Increased striatal DAT expression (IHC)	Domanskyi et al., 2011
TSC	*TSC1, TSC2*	Tsc1fl/fl x DAT-Cre	deletion	conditional KO	embryonic	P60-P112	No changes in SNc or VTA (IHC)	Increased soma size and dendritic branching in SNc and VTA (IHC)	Decreased excitability in SNc and VTA neurons	Hypertrohic axon terminals, greater enlargement in dorsal than ventral striatum (EM)	Strongly decreased evoked release, more pronouced in dorsal than ventral sitratum (FCV, electrical stim)	Increased in midbrain and striatum (IHC, WB)	Increased in dorsal and ventral striatum (HPLC)	Increased DAT function (FCV)	Kosillo et al., 2019

*Cre-ERT, tamoxifen-dependent Cre-recombinase; DA, dopamine; DAT, dopamine active transporter; EM, electron microscopy; FCV, fast-scan cyclic voltammetry; HPLC, high performance liquid chromatography; KO, knock-out; MFB, medial forebrain bundle; NAc, nucleus accumbens; PFC, prefrontal cortex; SNc, substantia nigra pars compacta; TH, tyrosine hydroxylase; VTA, ventral tegmental Area; WB, western blot; IHC, immunohistochemistry.*

#### *UBE3A*, Angelman Syndrome and ASD

*UBE3A* encodes an E3-ubiquitin ligase, which is part of the ubiquitin proteasome pathway that adds ubiquitin molecules to proteins that are destined to be degraded. In neurons, *UBE3A* is expressed from the maternal allele due to imprinting (Rougeulle et al., 1997; Vu and Hoffman, 1997). Loss-of-function mutations in the maternal *UBE3A* copy or *de novo* deletions of chromosomal region 15q11-q13, in which *UBE3A* resides, cause the severe neurodevelopmental disorder Angelman syndrome (AS), characterized by abnormal motor development, lack of speech, seizures, and a happy demeanor (Kishino et al., 1997; Matsuura et al., 1997). In turn, duplications of *UBE3A* or the 15q11-q13 locus are strongly associated with ASD, although small deletions have also been reported in ASD probands (Nurmi et al., 2001; Glessner et al., 2009; Hogart et al., 2010). *UBE3A* is a high confidence ‘score 1’ autism gene with 22 possible genetic variants reported.

There are several indications that *UBE3A* mutations could cause perturbations to the dopaminergic system. First, *Ube3a* is highly expressed in midbrain DA neurons in mice (Gustin et al., 2010). Second, the ubiquitin-proteasome degradation pathway is critically important for DA neuron function with aberrations in this system linked to the pathogenesis of both familial and sporadic forms of Parkinson’s disease (Olanow and Mcnaught, 2006). Third, patients with AS present with movement or balance disorders, usually ataxia of gait and/or tremulous movement of limbs, and frequently have rigidity and bradykinesia (Clayton-Smith and Laan, 2003). Reminiscent of Parkinson’s disease, which is caused by the degeneration of DA neurons, case reports suggest that resting tremor and rigidity in AS patients can be alleviated with the drug levodopa, a precursor for DA (Harbord, 2001) (although see also Tan et al., 2017).

Multiple studies have examined the dopaminergic system in a mouse model of AS that lacks the maternal copy of *Ube3a* (*Ube3a*^*m–/p*+^) (Jiang et al., 1998). An initial study reported that 7-8 month old male mice with loss of *Ube3a* had a reduced number of DA neurons in the SNc, but no change in striatal TH expression (Mulherkar and Jana, 2010). In contrast, using the same mouse model, another study in 3-4 month old male mice found no changes in midbrain DA neuron number or TH levels in dorsal or ventral striatum (Riday et al., 2012). Together, these data suggest a possible age-dependent decline in SNc DA neurons with loss-of-function of the maternal copy of *Ube3a*.

Electrophysiology recordings of VTA DA neurons in adult 2-3 month old *Ube3a*^*m*−/*p*+^ mice showed no changes in their intrinsic excitability (Berrios et al., 2016); however, SNc neurons were not assessed. Several studies have measured DA release in *Ube3a^*m–/p*+^* mice, with mixed results. Using *in vivo* fast-scan cyclic voltammetry (FCV), researchers observed increased electrically evoked DA release in the ventral striatum, but decreased release in the dorsal striatum in 3-4 month old male animals (Riday et al., 2012). Based on these results, it is attractive to speculate that AS-associated phenotypes of motor difficulties and happy disposition are generally congruent with reduced DA release in dorsal striatum and enhanced DA release in ventral striatum reported for the *Ube3a^*m–/p*+^* mouse model (Riday et al., 2012). That said, a later study found no changes in optogenetically evoked DA release in the nucleus accumbens in 2-3 month-old *Ube3a^*m–/p*+^* mice or in mice with maternal deletion of *Ube3a* in TH+ neurons only (Berrios et al., 2016). Despite no changes in DA release, the authors did observe a large decrease in GABA co-release from TH+ VTA neurons with maternal deletion of *Ube3a*.

In terms of DA production, while the study by Riday et al. did not find any differences in DA levels in the striatum or NAc measured by HPLC, another study reported a different result. In this study the authors compared *Ube3a^*m–/p*+^* mice with mice harboring a 6.3 Mb duplication of the conserved linkage group on mouse chromosome 7, which is equivalent to human chromosome 15q11-q13 (Nakatani et al., 2009). Increased striatal DA levels were detected in animals with maternal deletion of *Ube3a*, and either paternal or maternal duplication of the 15q11-q13 locus (Farook et al., 2012). Further work will be needed to understand at a mechanistic level how both deletion and duplication of *Ube3a* could increase striatal DA tissue content.

The maximum rate of DA re-uptake back into the pre-synaptic terminal, which is a key factor that controls extracellular DA levels, is determined by DAT availability and its substrate affinity. Several lines of evidence suggest that *Ube3a* loss impacts DAT function. For example, superfusion experiments of synaptosomes showed significantly increased basal efflux of [^3^H]MPP+ in *Ube3a^*m–/p*+^* mice compared to wild-type (Steinkellner et al., 2012). [^3^H]MPP+ is taken up selectively by DAT, and hence enables assessment of DAT function. Increased basal efflux of [^3^H]MPP+ suggests that with maternal *Ube3a* loss, extracellular DA levels could be increased, consistent with the findings of Farook et al. In the same synaptosomal preparation, researchers observed impaired [^3^H]MPP+ efflux in response to amphetamine (Steinkellner et al., 2012), which augments extracellular DA by reverse transport. Together, these observations indicate that DAT function is altered in *Ube3a^*m–/p*+^* mice. This conclusion is further supported by the observation that *Ube3a^*m–/p*+^* mice have reduced behavioral sensitivity to cocaine (Riday et al., 2012), a drug that enhances extracellular DA availability by blocking DAT function.

Overall, research published to date suggests that maternal loss of *Ube3a* has a significant impact on several aspects of DA neuron function. Specifically, there is a possibility of an age-dependent decline in SNc DA neuron number, a reduction in evoked DA release in the dorsal striatum, altered striatal DA tissue content, and aberrant DAT function when *Ube3a* expression is disrupted. The augmented evoked DA release in the ventral striatum of *Ube3a^*m–/p*+^* mice may involve non-cell autonomous factors (Sulzer et al., 2016), as it was observed *in vivo* but was not replicated with cell type-specific optogenetic stimulation of DA release in slices. It would be interesting for future studies to assess whether restoration of dopaminergic function is sufficient to improve behavioral phenotypes in mice with altered *Ube3a* expression.

#### *FMR1* and Fragile X Syndrome

Mutations in the X chromosome gene *FMR1*, which encodes the fragile X, which encodes the fragile X mental retardation protein (FMRP), cause Fragile X Syndrome (FXS), characterized by intellectual disability, physical features, and behavioral conditions. Up to ∼50% of male and ∼20% of female individuals with FXS are diagnosed with ASD (Bailey et al., 2008; Kaufmann et al., 2017). Currently, FXS is the leading monogenetic cause of ASD. FMRP has multiple functions but is most well known as an RNA binding protein that represses the translation of specific mRNAs (Darnell and Klann, 2013; Banerjee et al., 2018). The mRNAs bound by FMRP encode synaptic proteins as well as other proteins implicated in ASD risk (Darnell et al., 2011; Ascano et al., 2012). *FMR1* mutations include a trinucleotide CGG repeat expansion in the 5’ untranslated region (UTR) (Farzin et al., 2006; Clifford et al., 2007; Tassone et al., 2012). The full mutation exceeding 200 repeats is thought to result in abnormal methylation, which effectively silences *FMR1* expression (Pieretti et al., 1991; Primerano et al., 2002), while the premutation range of 55-200 CGG repeats typically causes increased *FMR1* mRNA expression (Kenneson et al., 2001; Peprah et al., 2010). *FMR1* is a high confidence ‘score 1’ autism gene with 35 possible genetic variants associated with ASD.

In terms of dopaminergic signaling, FMRP has been shown to be critical for the ability of D1-type DA receptors to exercise their neuromodulatory function in the prefrontal cortex (Wang et al., 2008; Wang et al., 2010; Paul et al., 2013). FMRP also serves as a mediator of cocaine-induced behavioral and synaptic plasticity in the NAc (Smith et al., 2014). Further, there are several lines of evidence suggesting that FMRP directly controls DA neuron function. First, FMRP is highly expressed in SNc neurons (Zorio et al., 2017). Second, there are several reports documenting the occurrence of Fragile X-associated tremor/ataxia syndrome (FXTAS) in adults over 50, particularly men, who carry an expanded *FMR1* allele in the premutation range (55-200 CGG repeats) (Leehey et al., 2007; Hall et al., 2009; Hall et al., 2010). These individuals present with a progressive neurodegenerative disorder characterized by kinetic tremor, ataxia and parkinsonism, although they typically do not meet the diagnostic criteria for Parkinson’s disease. Notably, a recent report showed that FMRP protein is lost in the SNc of Parkinson’s disease patients, and is further down-regulated as a result of Parkinson’s-linked α-synuclein overexpression in cultured DA neurons and mouse brain (Tan et al., 2019). Together, these studies indicate that changes in dopaminergic function may occur when *FMR1* expression is altered.

There are several animal models of FXS but the most widely used is the *Fmr1*^–/y^ mouse, which has a disrupted coding sequence resulting in loss of FMRP protein expression (Bakker et al., 1994; Kazdoba et al., 2014). In 12-16-week-old adult male mice, researchers demonstrated a significant reduction in the number of DA neurons in the SNc but not the VTA of *Fmr1*^–/y^ mice, with no overall change in TH levels in the dorsal or ventral striatum (Fish et al., 2013). The latter observation was replicated in a study showing normal TH expression in the striatum and midbrain in 8-16 week old *Fmr1*^–/y^ mice (Chao et al., 2020). However, it was observed that TH-positive dopaminergic axons in the striatum appeared more branched (Chao et al., 2020). This may indicate a compensatory change, as experimentally induced death of SNc DA neurons leads to increased axonal sprouting of the remaining dopaminergic cells (Tanguay et al., 2021). Together, these studies indicate that nigrostriatal DA neurons are particularly sensitive to loss of FMRP, with the surviving SNc DA neurons undergoing dynamic remodeling to increase axonal arborization and potentially boost TH expression to augment dopaminergic signaling.

In terms of dopaminergic output, Ventura and colleagues showed using *in vivo* microdialysis that there were no differences in basal DA levels in *Fmr1*^–/y^ mice in the prefrontal cortex but there was significantly reduced basal DA efflux within the striatum (Ventura et al., 2004). Reduced striatal DA transmission was also observed by FCV experiments in acute striatal slices, which showed that in *Fmr1*^–/y^ mice, evoked DA release was significantly diminished at 15-20 weeks of age, but not at 10 weeks of age (Fulks et al., 2010). Together these studies suggest that nigrostriatal-projecting SNc neurons may have reduced output when FMRP expression is disrupted and that this change may be age-dependent.

Changes in DAT function have also been observed in mouse models of FXS. Functional assessment of striatal DA re-uptake by FCV revealed an age-dependent decrease in DAT activity in adult *Fmr1*^–/y^ mice (Fulks et al., 2010). Another group verified decreased striatal expression of DAT in 8-12 week old *Fmr1*^–/y^ mice with western blot (Chao et al., 2020). The observed changes in DA release and re-uptake are not likely due to altered DA availability as total striatal tissue DA content does not appear to be consistently altered either in juvenile (4-6 week old) or adult (10-30 week old) *Fmr1*^–/y^ mice (Gruss and Braun, 2001, 2004; Fulks et al., 2010; Sørensen et al., 2015).

In summary, constitutive *Fmr1* deletion causes DA neuron loss that is specific to SNc neurons. The remaining DA neurons upregulate their TH expression such that there is no overall change in total striatal tissue DA content. Nonetheless, there is a significant age-dependent decrease in evoked DA release in the striatum, accompanied by reduced DA re-uptake due to a reduction in DAT expression. Both downregulation of DAT and increased axonal sprouting are likely compensatory changes, which attempt to augment DA signaling by the remaining SNc DA neurons. Thus, loss of FMRP appears to reduce dopamine signaling within the striatum; however, dopaminergic cells projecting to other regions, such as the prefrontal cortex, may be differentially affected.

#### *MECP2* and Rett Syndrome

Mutations in the MECP2 gene, located on the X chromosome, cause the severe neurodevelopmental disorder Rett syndrome (RTT) (Amir et al., 1999), which is characterized by progressive loss of motor and language functions, breathing problems, seizures, intellectual disability and autistic-like behaviors (Hagberg et al., 2002; Jeffrey et al., 2010). RTT affects 1/15,000 girls (Laurvick et al., 2006), although reports of male *MECP2* mutation carries have emerged recently (Reichow et al., 2015; Chahil et al., 2018; Pitzianti et al., 2019). MeCP2 protein has several proposed functions including in RNA splicing control and transcriptional regulation via binding to methylated DNA (Nan et al., 1997; Nan et al., 1998; Chahrour et al., 2008; Ip et al., 2018; Qiu, 2018). Importantly, *MECP2* mutations which both increase (e.g., duplication) and decrease (e.g., loss-of-function) its function are strongly implicated in neurodevelopmental disorders, including ASD and RTT (Carney et al., 2003; Samaco, 2004; Van Esch et al., 2005; Ramocki et al., 2010; Wang et al., 2016; Wen et al., 2017). There is significant variability in the clinical presentation of *MECP2* mutation carriers, with some types of mutations conferring milder phenotypes (Bebbington et al., 2008; Neul et al., 2008). Overall, *MECP2* mutations are strongly associated with ASD (Loat et al., 2008), and currently it is a high confidence ‘score 1’ autism gene with 180 possible genetic variants associated with ASD.

The importance of *MECP2* for dopaminergic function is supported by observations of low levels of monoaminergic metabolites and monoamine content in the cerebrospinal fluid of RTT patients (Roux and Villard, 2009; Samaco et al., 2009). In girls with RTT syndrome there are also reports of increased D2 receptor density in the brain (Chiron et al., 1993) and reduced DAT expression in the caudate-putamen (Wenk, 2007), both of which indicate reduced DA neurotransmission. Furthermore, regressive changes in fine motor control are associated with hyperkinetic features in young RTT patients, followed by bradykinesia and postural aberrations in RTT individuals in their late 20s (Fitzgerald et al., 1990; Temudo et al., 2008); both phenotypes are suggestive of dopaminergic alterations. In mice, *Mecp2* is expressed widely throughout the brain, including in the midbrain (Pelka et al., 2005). *Mecp2*^–/y^ mice are a widely used mouse model with constitutive loss of *Mecp2* (Guy et al., 2001). Similar to human RTT patients, *Mecp2*^–/y^ mice show deterioration of movement coordination between 4 and 9 weeks of age (Panayotis et al., 2011b; Kao et al., 2013; Liao, 2019). In turn, preservation of MeCP2 function selectively in TH-Cre-expressing catecholaminergic neurons is sufficient to prevent motor disturbances and other phenotypes (Lang et al., 2013).

Starting at 5 weeks of age, *Mecp2*^–/y^ mice present with a significant reduction in the number of SNc DA neurons and have reduced SNc neuron soma size (Panayotis et al., 2011b). Reductions in SNc neuron soma size and dendritic arborization have also been reported in female *Mecp2*^+/–^ mice and notably, these changes can be observed in the pre-symptomatic stage at 3-4 weeks of age (Gantz et al., 2011). Consistent with SNc cell loss, decreased TH expression in the midbrain and striatum of *Mecp2*^–/y^ mice has been observed in several studies (Samaco et al., 2009; Kao et al., 2013). In the midbrain, a significant decrease in the activated form of TH, which is phosphorylated at Ser40 (pSer40-TH), is first detectable at 5 weeks of age, and levels decline further by 8 weeks (Panayotis et al., 2011b). These studies point to a significant reduction in the DA synthesis capacity of the remaining SNc neurons in *Mecp2*^–/y^ mice. Consistent with this conclusion, several studies report a substantial reduction in total tissue DA content in the striatum (Panayotis et al., 2011a, b; Kao et al., 2013), with some also finding a significant reduction in total tissue DA content in the ventral midbrain (Kao et al., 2013). Given that these changes can be observed prior to symptom onset, this suggests that changes in nigrostriatal DA may be causal for the behavioral changes in *Mecp2*^–/y^ mice, particularly related to motor function (Liao, 2019). Yet not all studies replicate the reduction in total striatal DA content in this model. One report documents no alterations in DA or its primary metabolite DOPAC at 3 or 8 weeks of age in striatum or cortical regions, yet finds substantial and progressive decline in cerebellar DA content (Santos et al., 2010). The latter finding suggests possible cerebellar contribution to the motor phenotypes in RTT.

On balance, the evidence for reduced DA synthesis and content in mouse models of RTT is strong, and consistent with the finding that evoked DA release from SNc axon terminals in the dorsal striatum is decreased to just under 50% of control levels in *Mecp2^+/–^* female mice (Gantz et al., 2011). Impaired dopaminergic transmission in *Mecp2*^+/–^ animals is also supported by findings of decreased D2 autoreceptor current density in SNc cell bodies (Gantz et al., 2011). Since D2 autoreceptors normally constrain DA synthesis and release, a reduction in the D2R-generated current suggests a potential homeostatic alteration whereby the D2-mediated suppression of DA release is reduced in an attempt to boost DA signaling. Therefore, in *Mecp2*^+/–^ mice, DA transmission is compromised both at the axon terminals in the striatum and within the somatodendritic compartment in the midbrain. Consistent with this, augmentation of DA signaling with combined administration of levodopa and a Dopa-decarboxylase inhibitor is able to improve motor phenotypes and increase the life-span of *Mecp2*^–/y^ mice (Szczesna et al., 2014).

The reduction in total tissue DA content in *Mecp2*^+/–^ mice appears more pronounced in the rostral than caudal striatum (Kao et al., 2013). Emerging evidence suggests that there are regional specializations of striatal function and discrete computational circuits across the rostro-caudal axis, which in turn differentially control behavior (Hintiryan et al., 2016; Liao, 2019; Miyamoto et al., 2019). The observation of a rostral-to-caudal gradient of compromised DA synthesis was also found in *Mecp2*-conditional KO mice (Su et al., 2015) in which *Mecp2* deletion was restricted to forebrain GABAergic neurons. In this mouse model, researchers found that the DA tissue content was reduced in rostral striatum and increased in caudal striatum, with a concurrent reduction in pSer40-TH in rostral striatum and increase in caudal striatum (Su et al., 2015). Hence, GABAergic neurons lacking *Mecp2* appear to exert region-specific extrinsic control over local striatal DA production. Interestingly, conditional deletion of *Mecp2* from DA neurons using TH-Cre mice (Lindeberg et al., 2004) also leads to reduced TH expression and DA tissue content (Samaco et al., 2009). Therefore, multiple cell types may be involved in altered DA transmission in the context of *Mecp2* loss.

Overall, studies from mouse models show that disruption of *Mecp2* expression leads to a progressive loss of SNc DA neurons and a reduction in the soma size and dendritic branching of the remaining neurons, which is first observed in juvenile animals. The remaining SNc DA neurons have a diminished DA synthesis capacity and show a substantial reduction in dopaminergic transmission at striatal axon terminals and in the somatodendritic region. Furthermore, both cell autonomous and non-cell autonomous mechanisms result in compromised DA synthesis with *Mecp2* loss.

#### *PTEN* and *PTEN* Hamartoma Tumor Syndromes

*PTEN* encodes the phosphatidylinositol 3,4,5-trisphosphate 3-phosphatase and dual-specificity protein phosphatase PTEN (PTEN), which functions as a tumor suppressor by negatively regulating PI3K-AKT signaling (Maehama and Dixon, 1998; Stambolic et al., 1998). Loss-of-function mutations in *PTEN* cause a group of disorders known as PTEN hamartoma tumor syndrome (PHTS), which are characterized by benign tumors in peripheral organs and brain overgrowth (Hobert and Eng, 2009). *PTEN* mutations have been found in individuals with ASD and macrocephaly (head circumference >2 SD above the mean), with an estimated prevalence of 5-50% of *PTEN* mutation carriers meeting ASD diagnostic criteria (Butler et al., 2005; Buxbaum et al., 2007; Varga et al., 2009; Mcbride et al., 2010; Hansen-Kiss et al., 2017; Ciaccio et al., 2019). Several studies also find a strong association between *PTEN* mutations, including gene-disrupting *de novo* mutations, and ASD in individuals with typical brain/head growth (O’roak et al., 2012a, b; De Rubeis et al., 2014; Iossifov et al., 2015). Based on the evidence across multiple ASD patient cohorts, *PTEN* is classified as a high confidence ‘score 1’ autism gene with 129 possible genetic variants associated with ASD.

PTEN is involved in many processes relevant to brain development and neural circuit function via its regulation of PI3K-AKT and mechanistic target of rapamycin (mTOR) signaling (Van Diepen and Eickholt, 2008; Garcia-Junco-Clemente and Golshani, 2014; Skelton et al., 2019). By controlling cell cycle progression and cell migration/adhesion, PTEN is important for the proliferation and differentiation of neural progenitors (Groszer, 2001; Zhou and Parada, 2012; Tilot et al., 2015). In addition, PTEN controls neuronal size, dendritic morphology, and synaptic transmission (Fraser et al., 2008; Jurado et al., 2010; Sperow et al., 2012; Takeuchi et al., 2013). Specific to dopaminergic neurons, researchers have found elevated TH expression in the cortex and striatum of mice with a missense mutation in *Pten* (He et al., 2015). Additionally, cell type-specific deletion of *Pten* from DA neurons is sufficient to alter some aspects of social behavior (Clipperton-Allen and Page, 2014). Several studies demonstrated that loss of *Pten* protects dopaminergic cells from neurotoxic lesions and significantly enhances their survival (Diaz-Ruiz et al., 2009; Domanskyi et al., 2011). Furthermore, *Pten* suppression facilitates DA neuron integration in striatal grafts transplanted into a Parkinson’s disease mouse model (Zhang et al., 2012). Therefore, concurrent increases in AKT and mTOR signaling caused by *Pten* deletion enable increased TH expression and enhanced DA neuron survival under conditions of cell stress.

To investigate how cell autonomous loss of *Pten* affects the DA system, several mouse lines have been generated with either embryonic deletion of *Pten* from DA neurons using DAT-IRES-Cre mice or postnatal deletion using tamoxifen-inducible DAT-Cre mice. *Pten* deletion beginning at 8-10 weeks of age (Domanskyi et al., 2011) or embryonic *Pten* loss (Diaz-Ruiz et al., 2009) both resulted in a substantial increase in the soma size of SNc and VTA DA neurons and increased *Th* mRNA levels in the midbrain. Furthermore, both embryonic and adult *Pten* deletion caused increased TH protein expression in midbrain and striatum, with a concurrent increase in striatal DA tissue content (Diaz-Ruiz et al., 2009; Domanskyi et al., 2011). However, increased TH expression and striatal DA content had no effect on basal or evoked DA release or the rate of DA clearance by DAT examined with microdialysis (Diaz-Ruiz et al., 2009).

Some differences in the consequences of adult versus embryonic loss of *Pten* from DA neurons have been observed. For example, adult, but not embryonic, *Pten* deletion results in increased density of striatal DA axon terminals (Domanskyi et al., 2011). Mice with embryonic *Pten* deletion on the other hand, have an increased number of TH+ neurons in the SNc and VTA (Diaz-Ruiz et al., 2009; Inoue et al., 2013). This suggests that depending on the developmental timing, *Pten* loss promotes dopaminergic function either via increasing the numbers of DA neurons or enhancing axonal branching of DA neurons.

In summary, deletion of *Pten* leads to a substantial increase in DA neuron soma size in both the SNc and VTA and causes significant upregulation of TH expression and DA tissue content in the midbrain and striatum. This increase in DA synthesis and tissue content, however, does not appear to affect basal or evoked striatal DA release. Adult *Pten* deletion causes an increase in striatal DA axon density 6 weeks later, while embryonic loss does not appear to alter axonal arbors but increases the total number of TH+ neurons in the midbrain. Together these studies demonstrate that loss of *Pten* impacts several aspects of DA neuron structure and function, generally leading to DA neuron hypertrophy.

### *TSC1/2* and Tuberous Sclerosis Complex

*TSC1* and *TSC2* encode the proteins hamartin and tuberin, respectively. Hamartin and tuberin form a multimeric protein complex, which negatively regulates mTOR complex 1 (mTORC1) signaling (Tee et al., 2002; Huang and Manning, 2008). Loss-of-function mutations in either *TSC1* or *TSC2* cause Tuberous Sclerosis Complex (TSC), a neurodevelopmental disorder characterized by benign tumors in multiple organs, focal cortical malformations, epilepsy, and psychiatric and behavioral conditions (Krueger et al., 2013; De Vries et al., 2015; Henske et al., 2016). Between 25 and 50% of individuals with TSC are diagnosed with ASD, with greater prevalence in males (Smalley, 1998; Numis et al., 2011; Curatolo et al., 2015; De Vries et al., 2020). Importantly, mutations in *TSC1* and *TSC2* have also been reported in individuals with ASD independent of TSC (Schaaf et al., 2011; Esteban et al., 2012; O’roak et al., 2012a; Iossifov et al., 2015; Kalsner et al., 2018). Thus, both *TSC1* and *TSC2* are high confidence ‘score 1’ genes with 34 and 82 possible genetic variants, respectively, associated with ASD.

Several lines of evidence support the importance of mTORC1 signaling for DA neuron function. DA neurons have high metabolic demands to support their extensive axonal processes (Matsuda et al., 2009) and mTORC1 is a central regulator of cellular metabolism via its control of protein and lipid synthesis, mitochondrial biogenesis and autophagy (Saxton and Sabatini, 2017). Related to this, stimulation of mTORC1 signaling by expression of a constitutively active form of Rheb (caRheb), the small GTP-ase that directly promotes mTORC1 activity, protects SNc DA neurons from neurotoxin lesions (Kim et al., 2012). In turn, both acute inhibition of mTORC1 signaling with rapamycin (Hernandez et al., 2012) or 2 week-long *mTOR* deletion in adult VTA neurons (Liu et al., 2018) reduce evoked DA release in the dorsal and ventral striatum, respectively.

To examine the impact of constitutive mTORC1 activation on the dopaminergic system in the context of TSC, our group generated a DA neuron-specific *Tsc1* KO mouse (*Tsc1^*fl/fl*^; DAT-IRES-Cre*). Following embryonic loss of *Tsc1* and mTORC1 hyperactivation, no change in DA neuron cell number was observed (Kosillo et al., 2019). However, both SNc and VTA DA neurons had significantly increased soma size, as well as increased total length and number of dendritic arborizations (Kosillo et al., 2019). This morphological restructuring caused a significant reduction in DA neuron intrinsic excitability due to changes in passive membrane properties (Kosillo et al., 2019). TH protein expression in the striatum and midbrain and striatal DA tissue content were significantly increased (Kosillo et al., 2019). Despite increased DA synthesis and storage, evoked DA release assessed with FCV in striatal slices was severely compromised following *Tsc1* loss, with more profound deficits in the dorsal striatum compared to ventral regions (Kosillo et al., 2019). Electron microscopy analysis of striatal axon terminals showed significant enlargement of TH+ axon profiles, consistent with somatodendritic hypertrophy, which was most pronounced in the dorsal striatum (Kosillo et al., 2019). Axonal enlargement, in turn, was associated with greater vesicle distance from the plasma membrane and reduced vesicle clustering, which together likely reduces the efficiency of vesicle recruitment to release sites. Thus, the more pronounced axon terminal restructuring in the dorsal striatum is consistent with the larger DA release deficits in the dorsal versus ventral striatum. Notably, loss of Tsc1 from DA neurons did not impact motor or social behaviors but selectively impaired behavioral flexibility in a reversal learning task.

Importantly, some of the phenotypes observed in the Kosillo et al. study were also found in a postnatal model in which mTORC1 hyperactivation was caused by transduction of caRheb into SNc DA neurons at 8 weeks of age (Kim et al., 2012). 4 weeks post-injection, constitutive mTORC1 activation caused a significant increase in DA neuron soma size in the SNc and an increase in striatal TH expression, TH+ axon number, and DA tissue content (Kim et al., 2012), similar to postnatal loss of *Pten* (Domanskyi et al., 2011).

Overall, *Tsc1* loss leads to significant hypertrophy of SNc and VTA DA neurons at the level of soma, dendrites and axon terminals, while the total DA neuron number remains unchanged. Increased cell size, in turn, renders *Tsc1* KO dopaminergic cells hypoexcitable. *Tsc1* loss also results in significant upregulation of midbrain and striatal TH expression supporting elevated DA synthesis, with total striatal DA tissue content substantially increased. Despite this, evoked striatal DA release is compromised, at least in part due to aberrations in the architecture of axon terminals.

## Discussion

Across the five syndromic forms of ASD discussed here, there is clear evidence for alterations in the dopaminergic system that can include changes in DA neuron number, morphology, excitability, DA synthesis, and release. However, there is no consistent pattern of changes across different models. Soma size and DA synthesis capacity are impacted in all five syndromic ASD models, yet these can be changed in opposite ways. Alterations in cell number, axonal and dendritic architecture, membrane excitability and DA release were observed in some of the models; however, these properties were not universally assessed. Overall, findings from both syndromic and non-syndromic ASD mouse models support dopaminergic dysregulation being part of the molecular and cellular signature of autism in the brain (Robinson and Gradinaru, 2018). However, changes in DA function are not universal and are unlikely to be sufficient to drive all aspects of ASD.

The molecular processes controlled by UBE3A, FMRP, MECP2, PTEN and TSC1/TSC2 are important for maintaining balanced protein production and degradation and DA neurons may be particularly sensitive to aberrations in proteostasis. The studies discussed above show that alterations in protein degradation (*Ube3a*), gene expression (*Mecp2*), RNA regulation (*Fmr1*) or protein synthesis (*Pten, Tsc1*) can impact DA neuron structure and function. The overarching theme from these models is that interference with protein degradation or RNA processing leads to DA neuron death specifically in the SNc, coupled with progressive impairments in motor function. Specifically, gait, fine and gross motor coordination and motor learning are significantly compromised in both *Ube3a* and *Mecp2* mouse models (Jiang et al., 1998; Mulherkar and Jana, 2010; Panayotis et al., 2011b; Kao et al., 2013). Progressive motor decline is also seen in mice with *Fmr1* CGG expansion in the pre-mutation range (Van Dam et al., 2005). While not a core diagnostic feature of ASD, motor deficits are common in individuals with ASD (Fournier et al., 2010; Bhat et al., 2011; Esposito et al., 2011), and may even contribute to difficulties with speech acquisition. In turn, chronic increases in cellular anabolic processes do not cause degeneration of DA neurons but are sufficient to impair cognitive flexibility (*Tsc1*) (Kosillo et al., 2019) or social approach (*Pten*) (Clipperton-Allen and Page, 2014), which are related to core ASD symptoms. Further, there are indications of reduced sociability (Chao et al., 2020) and social discrimination (Sørensen et al., 2015) in *Fmr1* mouse models, while mice overexpressing *Ube3a* in the VTA exhibit sociability deficits that are precipitated by seizures (Krishnan et al., 2017). Together, these studies suggest that mutations in ASD-risk genes can alter dopaminergic function in a variety of ways, but that dopaminergic changes are not sufficient to recapitulate all aspects of ASD in a given mouse model.

Studies published to date have examined aspects of DA neuron function using a variety of metrics and approaches, and different experimental designs. Factors such as whether the gene disruption occurs embryonically or in adulthood, or constitutively or in a cell type-specific manner, could all contribute to differing results. Consequently, heterogeneity in methodology and outcome measures, such as whether DA release is measured by FCV or microdialysis, makes it challenging to make comparisons across different ASD models and studies. To formally address whether there are convergent changes in dopaminergic function in ASD, the optimal starting point would be assessment of the DA system in mice with a constitutive mutation, similar to patients, that show a wide range of ASD-related behavior phenotypes. Subsequently, the same mutation could be introduced selectively in DA neurons to define which dopaminergic signatures and behavioral phenotypes can be recapitulated with DA-specific manipulations.

Many of the findings discussed in this review come from *in vitro* and *ex vivo* assays that have provided fundamental insights into the state of the DA system in syndromic ASD mouse models. Recent developments in DA monitoring, including GRAB_*DA*_ and D-Light GPCR-based DA sensors (Labouesse et al., 2020; Patriarchi et al., 2020; Sun et al., 2020), coupled to spectral- and depth-resolved fiber photometry (Meng et al., 2018; Pisano et al., 2019), now grant unprecedented access to monitoring DA neurotransmission *in vivo*. The use of these newly developed tools to monitor DA dynamics in awake behaving animals, potentially across multiple brain regions simultaneously, will allow for comprehensive mapping of brain-wide DA neurotransmission dynamics and changes associated ASD. Furthermore, recently developed anatomical tools, which enable whole-brain pathway mapping via viral circuit tracing can be applied together with chemogenetic and optogenetic manipulations to aid in our understanding of ASD pathophysiology, as previously highlighted (Robinson and Gradinaru, 2018). Together, application of novel anatomical, circuit manipulating and DA monitoring tools will provide new insights into dopaminergic aberrations associated with ASD by connecting cellular and circuit-level aberrations with behavioral changes. This approach will facilitate the development of a data-driven theoretical frameworks on the role of DA in ASD pathogenesis.

In summary, as a neuromodulator, DA fulfills many distinct functions, and either too much or too little DA is likely to be disruptive to neural circuits with complex cascading effects. Some of these aspects may manifest early in development and over time exacerbate behavioral abnormalities culminating in ASD. In addition, dopaminergic changes associated with ASD are likely to be dynamic in nature, changing across the lifespan, and distinct between various brain regions and neural circuits. Consequently, further investigations are warranted to build a more complete picture of how DA dysregulation may contribute to ASD symptomology.

## Author Contributions

PK drafted the initial manuscript. HB contributed to the review and editing. Both authors devised the topic and scope of the review.

## Conflict of Interest

The authors declare that the research was conducted in the absence of any commercial or financial relationships that could be construed as a potential conflict of interest.

## Publisher’s Note

All claims expressed in this article are solely those of the authors and do not necessarily represent those of their affiliated organizations, or those of the publisher, the editors and the reviewers. Any product that may be evaluated in this article, or claim that may be made by its manufacturer, is not guaranteed or endorsed by the publisher.

## Footnotes

1 https://gene.sfari.org
